# Investigation of Causal Effect of Type 2 Diabetes Mellitus on Lung Cancer: A Mendelian Randomization Study

**DOI:** 10.3389/fgene.2021.673687

**Published:** 2021-08-31

**Authors:** Tongtong Hong, Na Qin, Xiaoyu Zhao, Cheng Wang, Yue Jiang, Hongxia Ma, Juncheng Dai

**Affiliations:** ^1^Department of Epidemiology, Center for Global Health, School of Public Health, Nanjing Medical University, Nanjing, China; ^2^Jiangsu Key Lab of Cancer Biomarkers, Prevention and Treatment, Collaborative Innovation Center for Cancer Personalized Medicine, Nanjing Medical University, Nanjing, China

**Keywords:** lung cancer, type 2 diabetes mellitus, Mendelian randomization, genome-wide association study, causation

## Abstract

**Background:**

Although several observational studies have attempted to investigate the association between type 2 diabetes mellitus (T2DM) and lung cancer risk, the results are controversial. Here, we intend to examine whether there is a causal association between T2DM and lung cancer risk.

**Materials and Methods:**

We conducted a Mendelian randomization (MR) study to systematically investigate the effect of T2DM on lung cancer among 13,327 cases and 13,328 controls. A weighted genetic risk score (wGRS) was constructed as a proxy instrument by using 82 previously reported T2DM-related single nucleotide polymorphisms (SNPs). The logistic regression model was utilized to estimate associations of T2DM-related SNPs and wGRS with lung cancer risk. Sensitivity analyses were also performed to assess the robustness of the observed associations.

**Results:**

We found no evidence for a causal relationship between T2DM and lung cancer risk (odds ratio, OR = 0.96, 95% confidence interval: 0.91–1.01, *p* = 0.96), and the association did not vary among populations of different age, sex, smoking status, and histological type. Sensitivity analyses (e.g., MR-Egger test) suggest that pleiotropic effects did not bias the result.

**Conclusion:**

In this MR study with a large number of lung cancer cases, we found no evidence to support the causal role of T2DM in lung cancer risk. Further large-scale prospective studies are warranted to replicate our findings.

## Introduction

Lung cancer is one of the most commonly diagnosed cancers and the leading cause of cancer-related death globally (Sung et al., 2021). It is estimated that 2.24 million new lung cancer cases and 1.8 million deaths occurred worldwide in 2020 (Sung et al., 2021). Tobacco consumption is recognized as the most critical risk factor for lung cancer, and approximately 90% of the cases can be attributed to tobacco exposure (Doll and Hill, 1950). Genetic factors also play an important role in the carcinogenesis of the lung. In the past decade, genome-wide association studies (GWASs) report 51 lung cancer susceptibility loci in different ethnic populations and highlight suspected causal genes at each locus (Bosse and Amos, 2018; Dai et al., 2019). However, the reported variants contribute to only 18% of the heritability of lung cancer (Mucci et al., 2016). The risk factors of lung cancer remain short of explored.

As a metabolic disease, the prevalence of type 2 diabetes mellitus (T2DM) is increasing worldwide, especially in Asia (Zheng et al., 2018). The International Diabetes Federation estimates that the age-adjusted comparative prevalence of T2DM among 20- to 79-year-olds in China was 9.7% in 2017 although the European region has a lower prevalence of about 6.8% (International Diabetes Federation, 2017). Because inflammation, insulin resistance, hyperinsulinemia, and hyperglycemia, which are biological alterations frequently observed in T2DM patients, may promote the initiation and progression of tumors, multiple studies have attempted to investigate the association of T2DM with cancer risk (Coussens and Werb, 2002; Arcidiacono et al., 2012; Ryu et al., 2014; Vigneri et al., 2016). Recently, several studies have also explored the association between T2DM and lung cancer risk, but no consistent conclusions have been drawn possibly due to the small sample sizes and confounding factors (Hall et al., 2005; Kuriki et al., 2007; Ogunleye et al., 2009; Ehrlich et al., 2010; Luo et al., 2012; Lo et al., 2013). In addition, for the existence of reverse causation, previous observational studies may not evaluate the causality accurately.

Mendelian randomization (MR) provides a novel approach to unbiasedly infer the causal relationship between exposure and outcome by using genetic variants as instrumental variables (IVs; Smith and Ebrahim, 2003), which can be quickly and accurately detected in large-scale epidemiological studies. This approach has been successfully applied to estimate the causal effect of polyunsaturated fatty acids and mosaic loss of chromosome Y on lung cancer (Wang et al., 2017; Qin et al., 2019). Recently, a large-scale meta-analysis (36,614 cases and 155,150 controls of Japanese ancestry) was conducted to evaluate the genetic influence on T2DM (Suzuki et al., 2019), providing us an opportunity to investigate the association between T2DM and lung cancer using the MR approach.

In this study, by using the genotype data of 26,655 participants (13,327 cases and 13,328 controls) with Asian ancestry, we derived a weighted genetic risk score (wGRS) with 82 T2DM-associated variants reported in Suzuki et al. (2019) as the IVs and applied the MR approach to investigate the causal relationship between T2DM and lung cancer.

## Materials and Methods

### Study Populations

In this study, a total of 13,327 cases and 13,328 controls from previously published lung cancer GWASs were included (Hu et al., 2011; Dai et al., 2019): (i) the Global Screening Array (GSA) Project of Nanjing Medical University (NJMU GSA Project with 10,248 cases and 9,298 controls) (Dai et al., 2019); (ii) the NJMU GWAS with 2,126 cases and 3,077 controls (Hu et al., 2011); and (iii) the NJMU OncoArray GWAS with 953 cases and 953 controls (Dai et al., 2019). Informed consent was obtained from all the participants included in this study, and each study was approved by the Ethics and Human Subject Committee of Nanjing Medical University. The basic characteristics of the included participants are summarized in Supplementary Table 1.

### Genotyping and Quality Control

Standard quality control processes were performed for each of these data sets to exclude unqualified samples and variants (Hu et al., 2011; Dai et al., 2019). Briefly, one single nucleotide polymorphism (SNP) was filtered out if it met one of the following criteria: (1) maps onto autosomal chromosomes, (2) had a call rate <95%, (3) had a minor allele frequency (MAF) in controls <0.005, or (4) showed a departure from Hardy–Weinberg equilibrium (HWE) in all samples (*p* ≤ 1 × 10^–5^) or deviated from HWE in the controls (*p*-value < 1.00 × 10^–7^) or cases (*p*-value < 1.00 × 10^–12^). We further excluded ineligible individuals if they (1) have overall genotype call rates less than 95%, (2) have gender discrepancies, (3) were duplicates or probable relatives (PI_HAT > 0.25), (4) have extreme heterozygosity rates (≥6 SD), or (5) were defined as outliers according to a principal component analysis (PCA) computed by EIGENSTRAT 3.0.

### Imputation

Imputation was performed for all data sets and has been described in our previous papers (Hu et al., 2011; Dai et al., 2019). Briefly, SHAPEIT V2 (Delaneau et al., 2011, 2013) was used to pre-phase the haplotypes. Then, IMPUTE2 (Howie et al., 2009) was used to impute ungenotyped SNPs to hg19 with the 1000 Genomes Project (Phase III integrated variant set across 2,504 samples^1^) as the reference. Poorly imputed variants with imputation quality score less than 0.40 were excluded from our analysis.

### Selection of T2DM-Related SNPs

Of the 88 T2DM-associated loci reported in Suzuki et al. (2019), six variants were excluded from further analysis: (1) five variants located on chromosome X and (2) rs77792157 with a *MAF* < 0.01 in all data sets. In addition, because indels were excluded in our imputation process, eight indels reported in Suzuki et al. (2019) were replaced by SNPs in high linkage disequilibrium (LD) (*r*^2^> 0.40) (Supplementary Table 2). The remaining 82 SNPs were independent and not in LD with each other (*r*^2^ < 0.10). The LD was estimated in the East Asian (EAS) population from the 1000 Genomes Project Phase 3 data set.

### Mendelian Randomization Estimates

A wGRS was constructed to predict T2DM for MR by multiplying the genotype dosage of 82 independent T2DM-increasing alleles with the following formula: $w\,G\,R\,S=\sum_{i=1}^{82}\beta_{i}\,S\,N\,P_{i}$, where βi is the beta coefficient of the *i*th SNP for T2DM from previous study (Suzuki et al., 2019) and *SNP*i is the dosage of the effect allele. We evaluated the association of genetically predicted T2DM and lung cancer risk in five data sets separately and then did a meta-analysis of these results. Associations of T2DM-related SNPs and wGRS with lung cancer risk were estimated by using a logistic regression model adjusted for age, sex, smoking status, and the first 10 principal components (PCs). Stratification analyses were performed based on age group (<60 and ≥60 years), gender, smoking status, and histology. In stratification analyses, a logistic regression model was also performed to evaluate the association with age, sex, smoking status, and the first 10 PCs as covariables when these were not the stratified factor. Cochran’s Q statistic was calculated to evaluate the heterogeneity between different subgroups.

### Pleiotropy and Sensitivity Analysis

In addition to the wGRS approach, the inverse-variance weighted (IVW) method with summary statistics of each genetic variant was also performed to assess the robustness of the association between genetically predicted T2DM and lung cancer risk (Burgess et al., 2013). The IVW regression function from the MR R package (Yavorska and Burgess, 2017) (0.4.1) was applied to evaluate the potential causal association between T2DM and lung cancer risk with T2DM-related SNPs as the IVs. Meanwhile, MR-Egger regression analysis was performed to evaluate the possible pleiotropic effect of this study’s genetic instruments (Bowden et al., 2015).

### Statistical Analysis

All statistical analyses were performed using PLINK (version 1.90) and R software (version 3.5.0). Two-sided *p*-values less than 0.05 were considered statistically significant.

## Results

### Study Populations

A total of 13,327 cases and 13,328 cancer-free controls of Asian ancestry were included in this study. Of the included participants, 51.60% (6,876/13,325) of the lung cancer cases and 51.67% (6,886/13,327) of the controls were over 60 years old. The majority (65.75%; 8,761/13,325) histological type of the included lung cancer cases was adenocarcinoma. Detailed demographics of subjects included in this study are shown in Supplementary Table 1.

### MR Analysis

**FIGURE 1:**
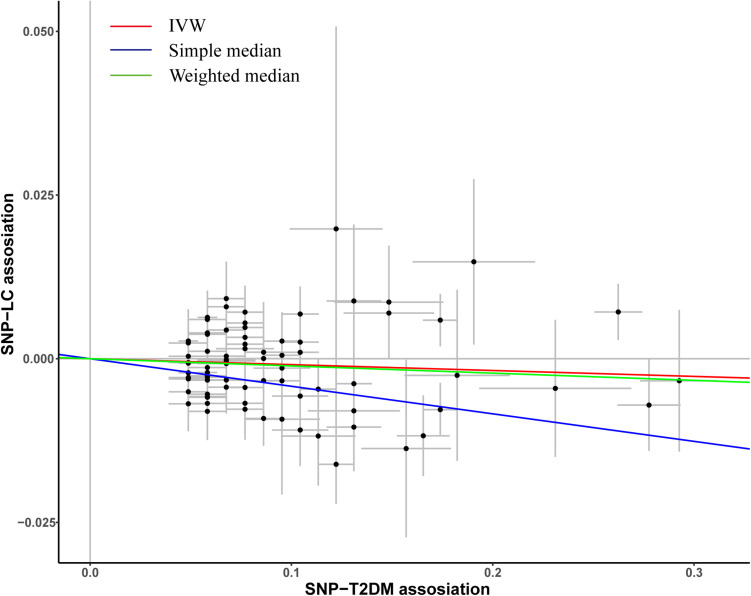
Plots of the effect size of each single nucleotide polymorphism (SNP) on T2DM and lung cancer risk. The x-axis plots the previously published β-estimate for the association of each SNP with T2DM. The y-axis plots the β-estimate from the multivariate logistic regression model for the association of each SNP with lung cancer risk in our study population. Lines represent causal estimates from the different methods.

**FIGURE 2:**
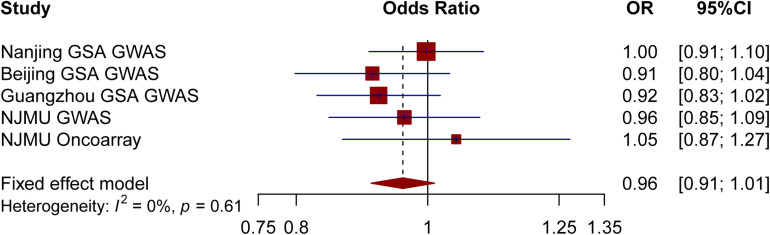
Risk of lung cancer for genetically predicted T2DM.

A total of 82 previously reported T2DM-related SNPs that achieved genome-wide significance (*p* ≤ 5.0 × 10^–8^) were included in the analysis. None of these SNPs had a significant association with lung cancer risk (*p* < 0.05/82), and the association between each variant with T2DM and risk of lung cancer is displayed in Figure 1. To evaluate the relationship between T2DM and lung cancer risk, we constructed a wGRS by using the genotype dosage of the abovementioned 82 SNPs. No statistically significant association was observed between genetically predicted T2DM and lung cancer [odds ratio (OR) = 0.96; 95% confidence interval (CI) = 0.91–1.01; *p* = 0.13; *P*_*heterogeneity*_ = 0.61; Figure 2] after adjusting for age, sex, smoking status, and the first 10 PCs.

### Subgroup Analyses

**FIGURE 3:**
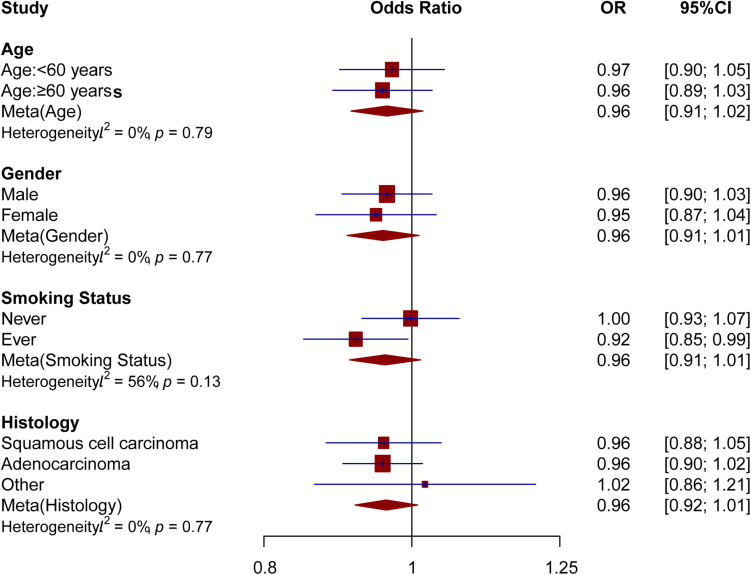
Stratified analyses of the association between T2DM and lung cancer risk.

Stratification analyses were also performed based on age, sex, smoking status, and histological type to evaluate whether the association between genetically predicted T2DM and the risk of developing lung cancer varies among different subgroups. As shown in Figure 3, the associations between T2DM and lung cancer risk were similar among subgroups divided by age, sex, smoking status, and histological type (*p*-values for heterogeneity were 0.79, 0.77, 0.13, and 0.77, respectively). However, for those who were ever smokers, we identified a marginally significant association between genetically predicted T2DM risk and lung cancer risk (OR = 0.92; 95%CI = 0.85–0.99; *p* = 0.03) although no significant association was observed in never smokers (*OR* = 1.00; 95%CI = 0.93–1.07; *p* = 0.96; *P*_*heterogeneity*_ = 0.13).

### Sensitivity Analysis

To evaluate the robustness of the observed association between genetically predicted T2DM and lung cancer risk, the IVW method was also conducted, and the association showed no difference with main results (OR = 0.99; 95%CI = 0.98–1.00, *p* = 0.13; Figure 1). Similar results were observed in simple median (OR = 0.99; 95%CI = 0.98–1.00, *p* = 0.13; Figure 1) and weighted median methods. The result of MR-Egger regression analysis suggests that no potential pleiotropic effect exists for variants used in constructing the genetic instruments (*p* = 0.85).

## Discussion

In this study, by using genotype data of 26,655 participants with Asian ancestry and 82 previously reported T2DM-related SNPs, we found no strong evidence to support the causal role of T2DM in lung cancer risk. This study is one of the largest MR analyses on T2DM and lung cancer risk to the best of our knowledge, and the findings are robust in sensitivity analyses with different methods.

Increasing evidence indicates the association between T2DM and cancer risk, and several plausible mechanisms underlying carcinogenesis are proposed (Giovannucci et al., 2010). Inflammation, insulin resistance, hyperinsulinemia, and hyperglycemia are the main pathophysiological characteristics of T2DM, which may be implicated in the pathogenesis of cancer among patients with T2DM (Kahn et al., 2014). Proinflammatory pathways can promote malignant transformation of cell carcinogenesis by inducing the production of inflammatory mediators, upregulating the expression of anti-apoptotic genes, and stimulating cell proliferation as well as angiogenesis (Landskron et al., 2014). Hyperglycemia can regulate cancer cell behavior, such as proliferation, migration, invasion, and recurrence, by causing DNA damage and activating various signaling pathways (Cencioni et al., 2014; Duan et al., 2014). Insulin resistance and hyperinsulinemia ultimately lead to elevated plasma insulin concentration, which may stimulate tumor growth by inducing the mitogenic effect and increasing bioavailable insulin-like growth factor 1 (Kazer, 1995; Chappell et al., 2001).

In the past decades, observational studies report somewhat inconsistent results regarding T2DM and lung cancer (Ehrlich et al., 2010; Rao Kondapally Seshasai et al., 2011; Hu et al., 2020). In the Nurses’ Health Study and the Health Professionals Follow-up Study with 3,814 lung cancer cases observed, incident T2DM was associated with an increased risk of lung cancer (Hu et al., 2020). In the retrospective cohort study with 1,811,228 participants, individuals with T2DM are at increased risk of several pulmonary conditions (asthma, COPD, fibrosis, and pneumonia) but not lung cancer (Ehrlich et al., 2010). However, these studies still cannot control the influence of potential biases because of the nature of the observational study. The present study provides no evidence to support a causal association between genetically predicted T2DM and lung cancer risk using an MR approach, which may control unmeasured confounders and reverse causation.

The strengths of our study include the large sample size with 13,327 lung cancer cases and the use of 82 independent T2DM-associated SNPs, which increases the statistical power of our study. By using individual genotype data of 26,655 participants, we were able to explore if there are any differences in the effect between different subgroups. Last, the consistency of findings across various MR methods, each based on different assumptions regarding pleiotropy, suggests that potential bias was unlikely to exist. Meanwhile, our study also has some limitations. Initially, it is difficult to avoid the influence of potential pleiotropy completely in any MR study, which may lead to biased causal effect estimates (Bowden et al., 2015). However, the pleiotropic effect was not observed in MR-Egger regression, and similar results were observed in sensitivity analyses using several other robust models. Furthermore, the findings were limited because T2DM-associated SNPs were derived from the Japanese population. For there is currently no large-scale T2DM GWAS study based on the Chinese population available.

In conclusion, we do not find clear evidence for a causal role of genetically predicted T2DM in the risk of lung cancer in a large, well-powered study, suggesting that previous associations between T2DM and lung cancer are possibly confounded by potential biases or due to reverse causation.

## Data Availability Statement

The datasets generated for this study are available on request to the corresponding author.

## Ethics Statement

The studies involving human participants were reviewed and approved by the Ethics Commission of the Nanjing Medical University. Written informed consent to participate in this study was provided by the participants’ legal guardian/next of kin.

## Author Contributions

TH, NQ, and JD contributed to the design and conception of the study. TH, NQ, and XZ did statistical analysis and wrote the manuscript. TH and XZ created the tables and figures. CW and YJ guided the manuscript writing. HM and JD corrected the manuscript draft and overall supervision of the project. All authors approved the final version of the manuscript.

## Conflict of Interest

The authors declare that the research was conducted in the absence of any commercial or financial relationships that could be construed as a potential conflict of interest.

## Publisher’s Note

All claims expressed in this article are solely those of the authors and do not necessarily represent those of their affiliated organizations, or those of the publisher, the editors and the reviewers. Any product that may be evaluated in this article, or claim that may be made by its manufacturer, is not guaranteed or endorsed by the publisher.

## Footnotes

1
http://www.internationalgenome.org/category/phase-3/

## Abbreviations

GWAS: genome-wide association study
T2DM: type 2 diabetes mellitus
MR: Mendelian randomization
IVs: instrumental variables
wGRS: weighted genetic risk score
GSA: Global Screening Array
SNP: single nucleotide polymorphism
MAF: minor allele frequency
HWE: Hardy–Weinberg equilibrium
PCA: principal component analysis
LD: linkage disequilibrium
PCs: principal components
IVW: inverse-variance weighted
OR: odds ratio
CI: confidence interval.
